# Synthesis and Characterization of Copolymers and Nanocomposites from Limonene, Styrene and Organomodified-Clay Using Ultrasonic Assisted Method

**DOI:** 10.3390/polym14142820

**Published:** 2022-07-11

**Authors:** Hodhaifa Derdar, Geoffey Robert Mitchell, Sarra Chaibedraa, Vidhura Subash Mahendra, Zakaria Cherifi, Khaldoun Bachari, Redouane Chebout, Fouzia Touahra, Rachid Meghabar, Mohammed Belbachir

**Affiliations:** 1 Centre de Recherche Scientifique et Technique en Analyses Physico-Chimiques (CRAPC), BP 10 384, Siège ex-Pasna Zone Industrielle, Bou-Ismail CP, Tipaza 42004, Algeria; hodhaifa-27@outlook.fr (H.D.); zakaria.cherifi.17@gmail.com (Z.C.); bachari2000@yahoo.fr (K.B.); rchebout@yahoo.fr (R.C.); tfafaze256@gmail.com (F.T.); 2 Laboratoire de Chimie des Polymères (LCP), Département de Chimie, FSEA, Oran1 University Ahmed Benbella BP N° 1524 El M’Naouar, Oran 31000, Algeria; sarachaibedraa@gmail.com (S.C.); rachidmeghabar@yahoo.fr (R.M.); legenial@gmail.com (M.B.); 3 Centre for Rapid and Sustainable Product Development, Institute Polytechnic of Leiria, 2430-080 Marinha Grande, Portugal; 4 School of Chemistry Food Science and Pharmacy, University of Reading, Reading RG6 6AD, UK; vidhumahendra@googlemail.com

**Keywords:** copolymerization, limonene, styrene, maghnite, nanocomposites

## Abstract

In the present work, we report a simple synthesis method for preparation of copolymers and nanocomposites from limonene and styrene using clay as a catalyst. The copolymerization reaction is carried out by using a proton exchanged clay as a catalyst called Mag-H^+^. The effect of temperature, reaction time and amount of catalyst were studied, and the obtained copolymer structure (lim-co-sty) is characterized by Fourier transform infrared spectroscopy (FT-IR), nuclear magnetic resonance spectroscopy (^1^H-NMR) and differential scanning calorimetry (DSC). The molecular weight of the obtained copolymer is determined by gel permeation chromatography (GPC) and is about 4500 g·mol^−1^. The (lim-co-sty/Mag 1%, 3%, 7% and 10% by weight of clay) nanocomposites were prepared through polymer/clay mixture in solution method using ultrasonic irradiation, in the presence of Mag-CTA^+^ as green nano-reinforcing filler. The Mag-CTA^+^ is organophilic silicate clay prepared through a direct exchange process, using cetyltrimethylammonuim bromide (CTAB). The prepared lim-co-sty/Mag nanocomposites have been extensively characterized by FT-IR spectroscopy, X-ray diffraction (XRD), scanning electronic microscopy (SEM) and transmission electronic microscopy (TEM). TEM analysis confirms the results obtained by XRD and clearly show that the obtained nanocomposites are partially exfoliated for the lower amount of clay (1% and 3% wt) and intercalated for higher amounts of clay (7% and 10% wt). Moreover, thermogravimetric analysis (TGA) indicated an enhancement of thermal stability of nanocomposites compared with the pure copolymer.

## 1. Introduction

The production of polymers based on renewable monomers has been the subject of several recent research groups around the world. Of the different types of polymers studied, those based on renewable resources have been the most extensively studied [1,2,3]. The most successful studies of terpene reactions in organic chemical synthesis have been published [4], but their applications in polymer science are still few. Limonene is a monocyclic terpene found in many essential oils extracted from citrus peels and has been used as a flavor and green solvent in cosmetics, food and beverages. Limonene is of particular interest in the field of polymerization because it contains double bonds that provide the difunctional groups required for polymerization. Limonene is also an allylic monomer (CH_2_=CH–CH_2_Y) [5,6]. A literature search reveals that chemists have attempted to develop a substitute for polyterpenes from petroleum distillates [7], but no substitute has yet been developed, as most terpenes are not homopolymerized by bulk steric [8], low stabilization energy between monomers and transition state radicals [9], with the exception of β-pinene and limonene which were polymerized by clay [10,11], Ziegler-Natta [12] as well as Friedel-Crafts catalysts [13]. Limonene is widely used in cosmetics and other products, as a food additive, medicine and even as a green solvent [14].

Styrene is one of the most studied and widely used monomers, both in industry and academia, as its polymerization is used as a model for chain polymerization [15]. Styrene is also utilized as a comonomer in copolymers synthesis, including acrylonitrile butadiene styrene (ABS) [16], rubber or latex based on styrene butadiene (SBS) [17], styrene-acrylonitrile (SAN) [18], acrylonitrile styrene acrylate (ASA) [19] and unsaturated polyesters. During World War II, the production of styrene, in particular, grew rapidly in the United States to meet the army’s demand for synthetic rubber. Styrene is also used in small amounts in perfumes and medications [20], as well as in the manufacture of polyester resins [21].

In recent decades, there has been an increasing interest in nanocomposites, a new class of materials reinforced by nanoparticles. Toyota researchers sparked interest in these novel materials in the early 1990s. In fact, they demonstrated a considerable increase in dimensional stability by dispersing clays in polyamide-6 by in situ polymerization [22]. These findings paved the way for new possibilities for polymer matrix nanocomposites in a variety of domains [23]. Nanocomposites made of toxic polymers have been phased out in favor of those made of green materials in recent years. The addition of a predetermined amount of clay as reinforcement to a polymer matrix improves the physicochemical properties of the resulting nanocomposites [24,25]. Two types of nanocomposites structures, intercalated and exfoliated nanocomposites, can be created based on the interaction strength of the modified polymer/clay. Nanocomposites can be made using different techniques, including in situ polymerization, polymer solution blending and other techniques [26].

Ultrasound is being used to prepare nanocomposites based on polymer and clay in solution, which is highly interesting. This method of production was employed to minimize reaction time and boost nano-filler dispersion in polymer matrix, with the majority of them focused on exfoliating the packed clay layers [27,28,29,30]. Ultrasonication induces acoustic stream and cavitation bubbles, which then undergo an implosion process, releasing heat and energy and resulting in a highly well dispersed reaction medium [31]. The use of ultrasound has an impact on the morphology of the prepared nanocomposites, especially in dispersion, such as in situ polymerization methods [32]. Several nanocomposites based on polymers and clay were created using an ultrasound-assisted technique for these reasons.

According to the literature, nanocomposites based on limonene, including poly (lactic acid)/D-limonene/ZnO bio-nanocomposites [33], Pt, Ru and Ni/graphene nanocomposites [34] and V-MCM-41 nanocomposites [35], but the use of natural clay as a catalyst in the synthesis of copolymers based on limonene and styrene is almost non-existent. The main goal of this research is to look into the catalytic properties of a natural clay (Mag-H^+^) as a new non-toxic catalyst for the copolymerization of limonene and styrene and Mag-CTA^+^ as a new nano-reinforcing filler for the fabrication of nanocomposites based on styrene-limonene copolymer (lim-co-sty) using an ultrasonic assisted approach to improve the copolymer’s thermal and mechanical properties. We previously showed the benefits of various uses of this catalyst type and the kind of nano-reinforcing filler in different polymerization reactions and nanocomposites synthesis in our published work [36,37,38].

## 2. Materials and Method

### 2.1. Materials

(R)-(+)-Limonene (97%), styrene (99%), methanol (CH_3_OH, 99.9%), dichloromethane (CH_2_Cl_2_, 99.8%), sulfuric acid (H_2_SO_4_), sodium chloride (NaCl) and cetyltrimethylammonuim bromide (CTAB) were purchased by Sigma Aldrich (St. Louis, MO, USA) and used as received without further purification. ENOF Bental Spa of the National Company of Nonferrous Mining Products, Maghnia Unit (Algeria), supplies Maghnite (Algerian montmorillonite clay) in its natural state. The ultrasound equipment used to prepare Mag-CTA^+^ and nanocomposites consists of a jacketed glass tank with an ultrasonic horn (13.6 mm diameter, non-replaceable tip composed of Titanium alloy Ti-6Al-4V) and a Sonics VC-750 Vibra 6 Cell generator (Sonics & Materials, Newtown, CT, USA).

### 2.2. Preparation of Maghnite-H^+^

A procedure identical to that described by Derdar et al. [39] was used to prepare Mag-H^+^. Raw-Mag was activated using a sulfuric acid solution to produce clay that has been exchanged with protons. Crushed raw Maghnite (30 g) was disseminated in distilled water in an Erlenmeyer flask (120 mL). A magnetic stirrer was used to agitate the mixture for 2 h at room temperature. Then a 0.5 M of sulfuric acid solution (100 mL) was added. The resulting mixture was kept under constant stirring for two days. The mineral was filtered and rinsed with distilled water several times until it reached a pH of 7. After filtration, Mag-H^+^ is dried in an oven at 105 °C for 24 h before being crushed.

### 2.3. Activation of Mag-Na^+^ and Mag-CTA^+^

Derdar et al. [40] established a procedure for preparing Mag-Na^+^. The raw-Mag given by Bental Spa was crushed and finely sieved, and the sodium activation of Maghnite was performed using 1 L of NaCl solution (1 M) and 20 g of raw-Mag (2% by weight), which was combined for 24 h at room temperature before being washed multiple times with distilled water. Ultrasound was used to activate Mag-CTA^+^ for one hour [41]. To begin, place 10 g of Mag-Na^+^ in a 1 L Erlenmeyer flask with the desired concentration (1 CEC). The suspension was filtered and rinsed many times with distilled water at the end of the exchange procedure. Finally, the solid was dried for 24 h at 105 °C and crushed. FT-IR and XRD study establish the structure of the organophylic clay, while SEM and TEM analysis investigate their morphological properties.

### 2.4. Copolymerization Procedure

**Scheme 1:**
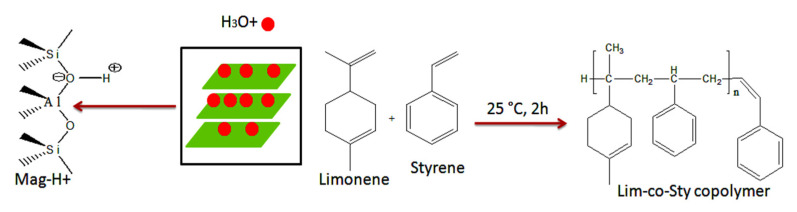
Copolymerization of limonene with styrene using Mag-H^+^.

The reaction was carried out in solution at room temperature, 0.02 mol of limonene and styrene, are kept under stirring for 2 h in 10 mL of CH_2_Cl_2_, with 10% by weight of Mag-H^+^ (Scheme 1). Table 1 summarizes the operating conditions of the copolymerization. After 2 h, the reaction mixture was filtered, precipitated in cold methanol and dried under vacuum overnight. The obtained copolymer was a solid product. Regarding the kinetic study, the same procedure described above was repeated by changing the percentage of the catalyst amount, time of the reaction and the temperature, in order to find the optimal reaction conditions.

### 2.5. Synthesis of Nanocomposites Copolymer/Clay (Lim-co-Sty/Mag)

**Scheme 2:**
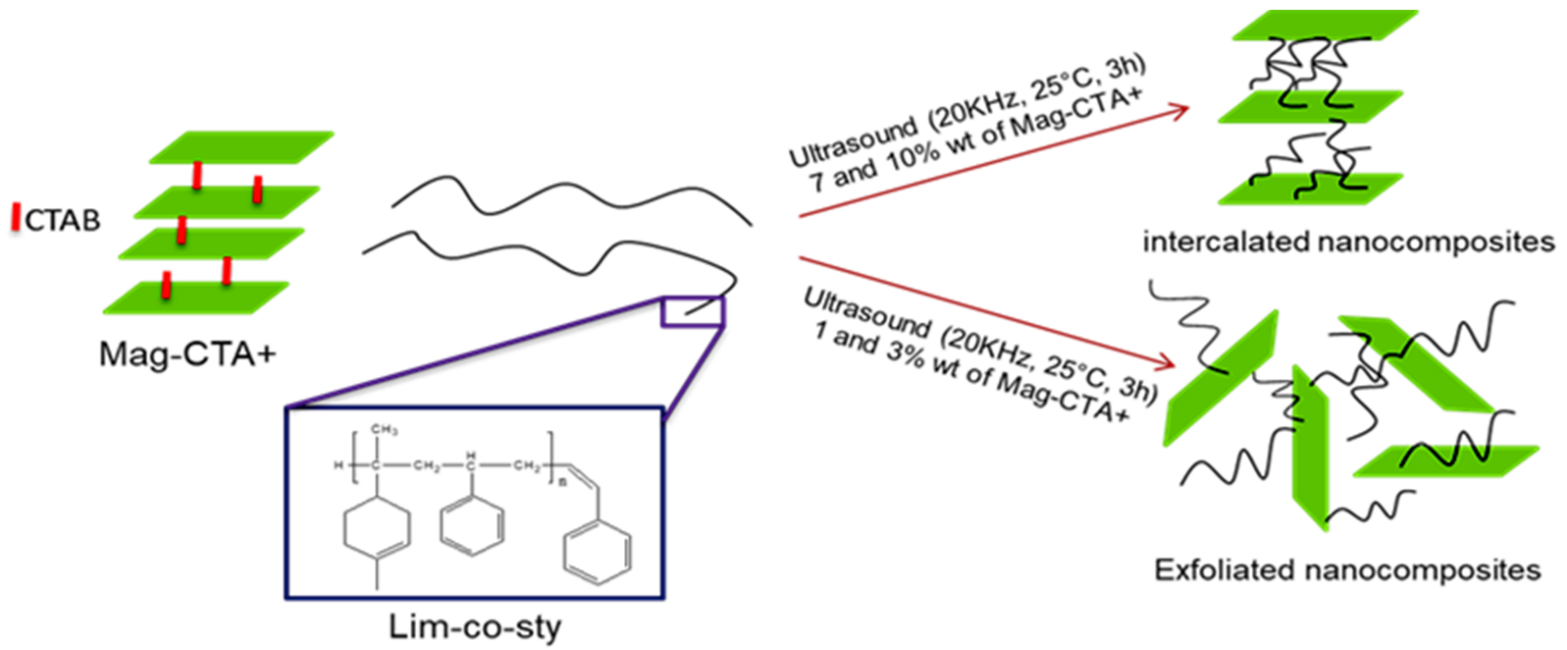
Synthesis of nanocomposites (Lim-co-sty/Mag) using ultrasonic irradiation.

The polymer/clay mixture in solution synthesis method was used to prepare Lim-co-Sty/Mag nanocomposites. Then, 1 g of the resulting copolymer (Lim-co-Sty) is dissolved in 25 mL of CH_2_Cl_2_. The copolymer was then thoroughly dissolved by stirring the solution for 15 min. After that, 1% by weight of Mag-CTA^+^ is added to the solution, and the mixture is treated for 3 h using an ultrasonic-assisted technique [42]. The nanocomposite was then precipitated in methanol, filtered and dried under vacuum overnight (Scheme 2). The same process was used by adding different amounts of Mag-CTA^+^ to Lim-co-Sty copolymer 3%, 7% and 10% by weight (see experimental conditions in Table 1).

### 2.6. Characterization

The functional groups of the resulted copolymer, modified clay and nanocomposites were studied using BRUKER ALPHA Diamond-ATR infrared spectroscopy (Bruker, Billerica, MA, USA) in the range of 4000–360 cm^−1^. ^1^H-NMR analysis in Deuterated Chloroform using Brucker-Avance 300 MHZ equipment, validate the structure of the obtained copolymer. Differential scanning calorimetry (DSC) was also used to study the thermal properties of Lim-co-Sty copolymer, by using calorimetric analysis (DSC) 204 F1, NETZSCH equipment (Selb, Germany), operating at a heating rate of 20 °C/min, from room temperature up to 450 °C under an inert atmosphere with a flow rate of 50 mL/min. The molecular weight of the prepared copolymer was studied by a GPC-PL120 apparatus, using CH_2_Cl_2_ (1.0 mL/min) as the mobile phase at 27.5 °C. Polystyrene standards are used for calibration. XRD analysis on a Bruker AXS D8 diffractometer (Cu-K radiation) and FEG-SEM on a JEOL 7001F electron microscope (JEOL, Tokyo, Japan) were used to examine the surface morphology of the modified clay and nanocomposites. A Hitachi 8100 (Hitachi, Tokyo, Japan) was used to take transmission electron micrographs. Thermogravimetric analysis (TGA) was performed under nitrogen with a PerkinElmer STA 6000 (PerkinElmer, Waltham, MA, USA) in the temperature range of 30–700 °C and a heating rate of 20 °C/min to determine thermal characteristics of the obtained nanocomposites.

## 3. Results

### 3.1. Characterization of the Modified Clay (H^+^, Na^+^ and CTA^+^)

**Figure 1:**
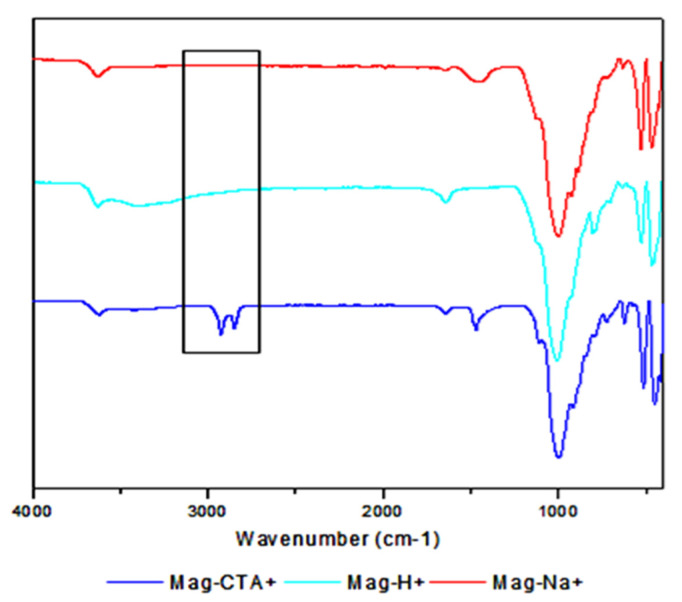
FT-IR spectra of Mag-H^+^, Mag-Na^+^ and Mag-CTA^+^.

Figure 1 shows the FT-IR spectra of Mag-H^+^, Mag-Na^+^ and Mag-CTA^+^. We see a strong peak at 1057 cm^−1^ and two bands at 455 cm^−1^ and 515 cm^−1^, which correspond to the Si–O–Si and Si–O–Al bonds’ elongation vibrations, respectively [43,44]. Following the modification of Maghnite by CTAB, two additional bands, corresponding to the C–H stretching vibrations of the methyl group, were found in the 2850 cm^−1^ and 2922 cm^−1^ areas for Mag-CTA^+^. According to the results of FT-IR analysis, the CTA^+^ alkyl ammonium ions intercalate between the clay sheets.

**Figure 2:**
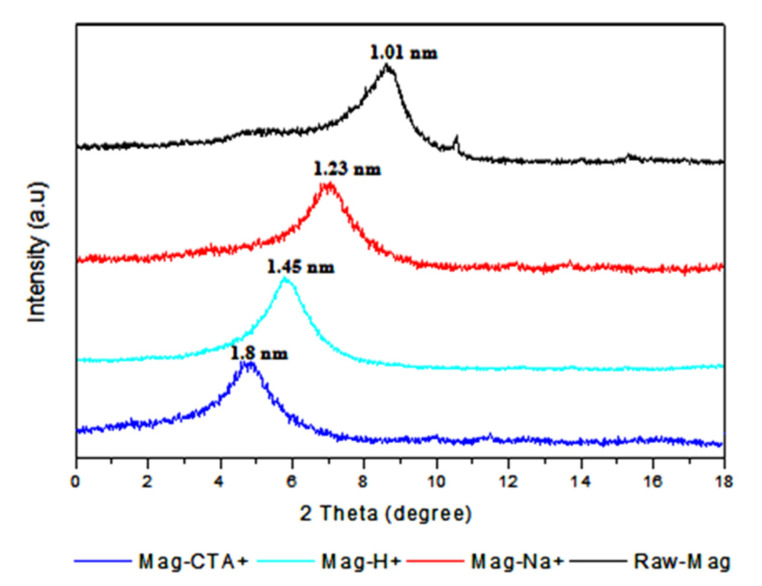
XRD patterns of Raw-Mag, Mag-H^+^, Mag-Na^+^ and Mag-CTA^+^.

Raw-Mag, Mag-H^+^, Mag-Na^+^ and Mag-CTA^+^ X-ray diffractograms are shown in Figure 2. We calculated basal spacing (d_001_) from XRD patterns using the Bragg equation (2.d.sinθ = n.λ), which is 1.01 nm for Raw-Mag and 1.45 nm for Mag-H^+^. The substitution of a single water layer between the sheets of Raw-Mag by two interlamellar water layers in Mag-H^+^ explains this increase in basal spacing. Mag-Na^+^ and Mag-CTA^+^ diffractograms have different basal spacing (d_001_), range from d = 1.23 nm for Mag-Na^+^ to d = 1.8 nm for Mag-CTA^+^. The intercalation of the CTAB’s alkyl ammonium ions in the inter-foliar galleries is confirmed by this increase. The influence of ultrasonic irradiation on the preparation of Mag-CTA^+^ can be seen in these data. Aicha Khenif et al. [45] obtained an interlayer distance of 1.98 nm after 24 h of stirring, but in our case, an interlayer distance of 1.8 nm was produced after only 1 h of stirring.

### 3.2. Characterization of the Obtained Copolymer (Lim-co-Sty)

#### 3.2.1. H-NMR Measurements

**Figure 3:**
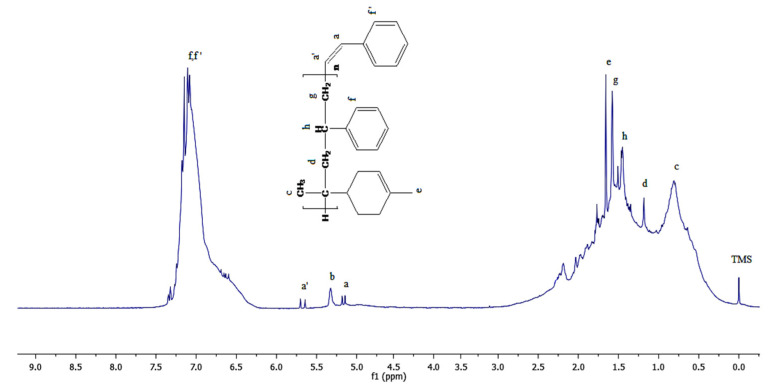
^1^H-NMR spectrum of the obtained copolymer (lim-co-sy).

**Figure 4:**
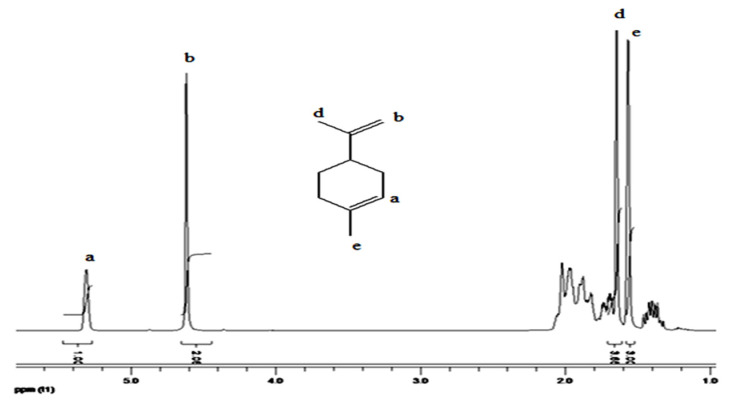
^1^H-NMR spectrum of limonene.

^1^H-NMR spectrum of the obtained copolymer is shown in Figure 3. The ^1^H-NMR spectrum was obtained to further investigate and confirm the proposed structure. ^1^H-NMR spectra of (lim-co-sty) clearly show a signal at 0.8 ppm as interfered several peaks corresponding to the protons of the methyl group. There is also the appearance of the peak (d) at 1.16 ppm in the spectrum of the obtained copolymer corresponding to the protons of methylene group (–CH_2_–), this peak does not appear in the spectrum of limonene. The peaks (a) and (a’) at 5.15 and 5.66 ppm are the characteristic resonance of the protons resulted by the terminal double bond (–CH=CH–) of the styrene. The peak (b) at 5.32 ppm is the characteristic resonance of the protons resulted by the internal double bond (C=C) of limonene. The comparison of ^1^H-NMR spectra of the copolymer (lim-co-sty) with those of limonene in Figure 4 shows that the peak b at 4.6 ppm due to disubstituted olefinic protons (C=CH_2_) of limonene has disappeared. These results show, clearly, that the copolymerization of limonene with styrene is successful with Mag-H^+^.

#### 3.2.2. FT-IR Measurements

**Figure 5:**
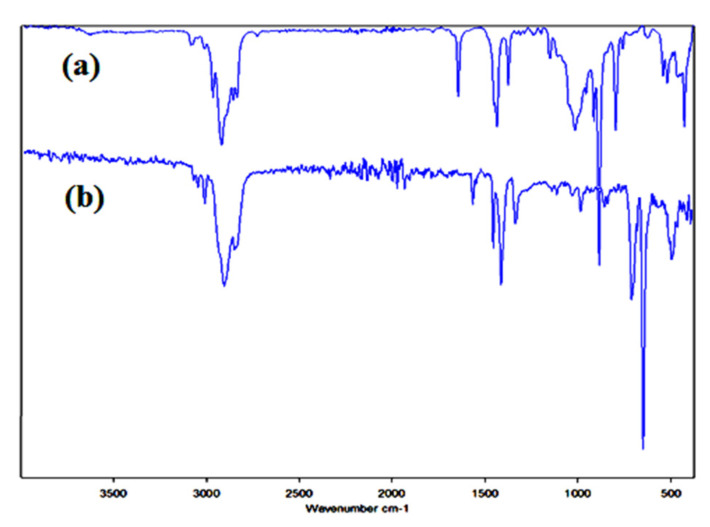
FT-IR spectra of limonene (**a**) and lim-co-sty (**b**).

The structure of the obtained copolymer was also confirmed by FT-IR measurements. FT-IR spectrum of limonene (a) and the obtained copolymer (b) have been shown in Figure 5. It is observed that the peaks at 1309 cm^−1^, 1217 cm^−1^, 956 cm^−1^, 913 cm^−1^ and 885 cm^−1^ corresponding to the double bonds in limonene have disappeared in the spectra of the copolymer confirming that the copolymerization has succeeded. It should be noted the presence of characteristic band corresponding to the stretching band of C=C at 1640 cm^−1^ in the spectra of limonene and at 1600 cm^−1^ in the spectra of lim-co-sty corresponding to the terminal double bond (–CH=CH–) of the styrene and, also, an intense band at 2930 cm^−1^ corresponding to the valence vibration of the methylene C–H. The bands at 1456 and 1365 cm^−1^ are attributed to deformation of C–H bond of the CH_2_ and CH_3_ groups. A band at 886.38 cm^−1^ corresponds to the valence vibration C–H bond of CH_2_ out-of-plane. The FT-IR spectrum of the obtained copolymer shows, also, the presence of an intense band at 600 cm^−1^ corresponding to the benzene cycle of styrene which confirms the results obtained by NMR analysis.

#### 3.2.3. Differential Scanning Calorimetry (DSC)

**Figure 6:**
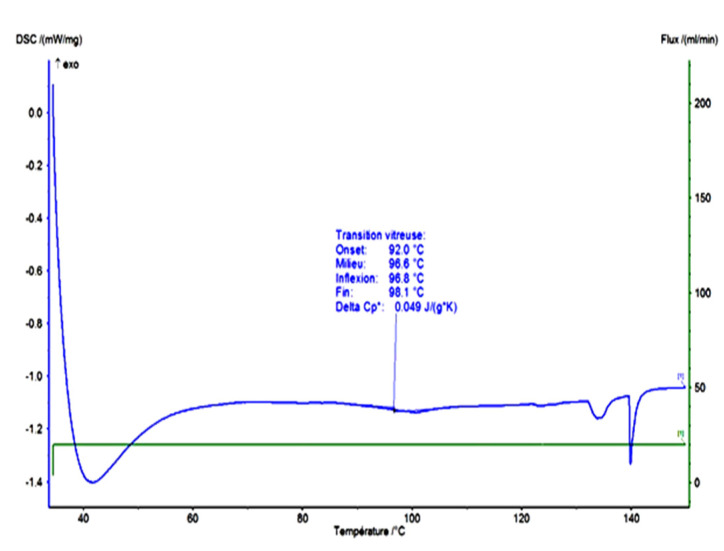
DSC curve of the obtained copolymer.

Differential scanning calorimetry (DSC) was used to study the thermal properties of the obtained copolymer. Figure 6 shows the DSC curve of lim-co-sty. The glass transition temperature (Tg) recorded from the DSC curve of the copolymer is observed in the temperature range of 92–98.1 °C. In addition, the comparison of the Tg of the copolymer with those of polystyrene (Tg about 100 °C) [46] and polylimonene (Tg = 116 °C) [47], shows clearly that the copolymerization of limonene with styrene using Mag-H^+^ as a catalyst is successful.

#### 3.2.4. GPC Measurements

**Figure 7:**
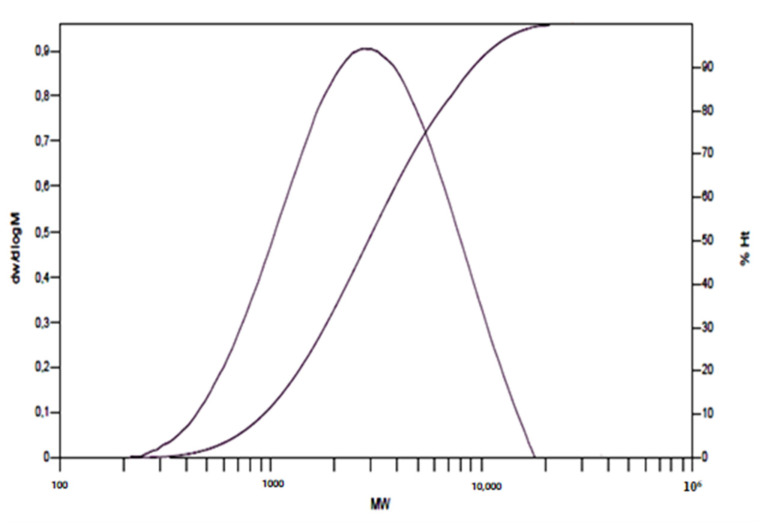
GPC chromatogram of the obtained copolymer.

The average molecular weight of the obtained copolymer Lim-co-sty was measured by GPC analysis. The results of GPC analysis are presented in Figure 7. GPC chromatogram shows that the molecular weight of the obtained polymer (Mn) is about 4410 g/mol, Mw = 4500 g/mol and Mw/Mn =1.02. This molecular weight is low. Comparing the molecular weight of copolymers based on styrene obtained by radical polymerization [48], but using Mag-H^+^ as a catalyst is still preferred for its many advantages such as very low purchase price compared to other catalysts and the easy removal of the reaction mixture.

### 3.3. Effects of Various Synthesis Parameters on the Copolymerization Yields

The objective of this part is to study the kinetic of the copolymerization of limonene with styrene by Mag-H^+^, which consists in varying separately different parameters including the catalyst amount, the reaction time and the temperature, in order to study their influence on the yield of the obtained product and to find the optimum conditions of the copolymerization reaction.

#### 3.3.1. Effect of Catalyst Amount

**Figure 8:**
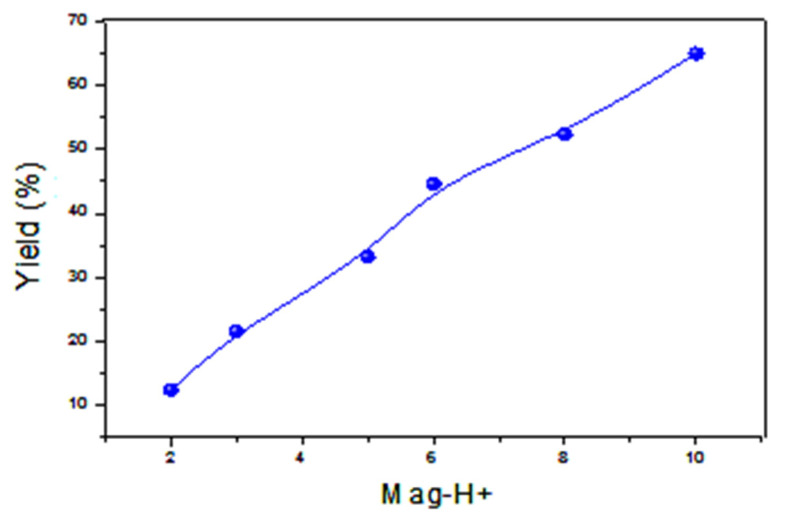
Effect of catalyst amount on copolymerization efficiency.

In order to find the optimal conditions for the copolymerization of limonene with styrene and to follow the effect of the quantity of the catalyst on the yield of the copolymerization, on this study we varied the catalyst/monomer (2%, 3%, 5%, 6%, 8% and 10% by weight of Mag-H^+^), this study was carried out at room temperature for 2 h. The obtained results are shown in Figure 8 and show that the yield increases with the increase in the catalyst amount, the best yield (65%) was obtained with 10% of Mag-H^+^. This phenomenon is essentially due to the number of active sites present in the reaction medium because these are proportional to the mass of the catalyst. These active sites are responsible for the initiation and acceleration of the polymerization reaction until there saturation.

#### 3.3.2. Effect of the Temperature

**Figure 9:**
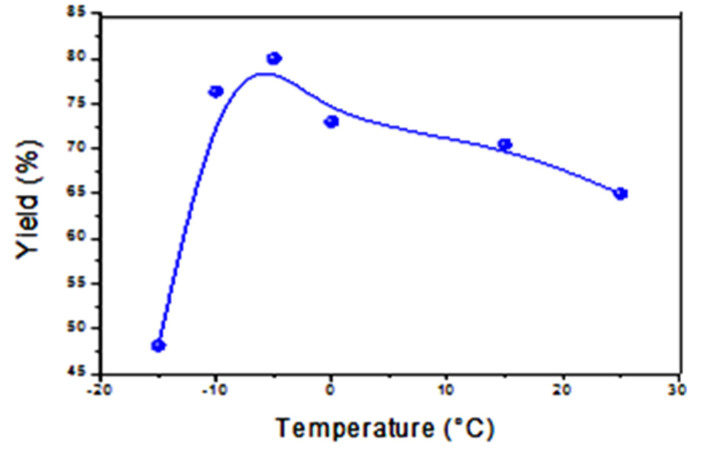
Effect of temperature on copolymerization efficiency.

In this study, the copolymerization reactions were carried out for 2 h using 10% by weight of Mag-H^+^. Figure 9 shows the effect of temperature on the yield of the copolymerization. This study is carried out in solution at different temperatures: −15 °C, −5 °C, 0 °C, 15 °C, 15 °C and 25 °C. An interesting result is that Mag-H^+^ is able to initiate the copolymerization even at low temperature with CH_2_Cl_2_. The yield of the reaction reaches its maximum (80%) for a temperature of −5 °C. The yield decreases with the temperature from 0 to 25 °C. It should be noted that the temperature has a great influence on the yield of the copolymerization reaction.

#### 3.3.3. Effect of Reaction Time

**Figure 10:**
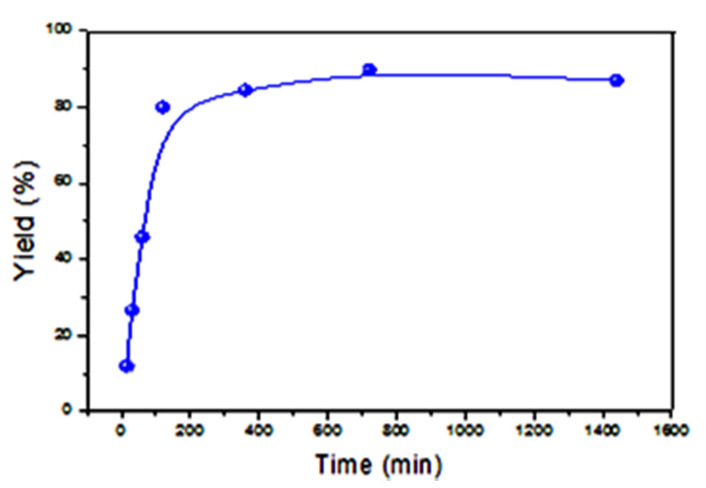
Reaction time effect on the copolymerization yield.

The influence of time on the yield of copolymerization of limonene with styrene was studied at −5 °C in CH_2_Cl_2_ with 10% Mag-H^+^. Figure 10 shows the effect of reaction time on the yield of the obtained copolymer. As shown in Figure 10 after 6 h, the copolymerization proceeds rapidly and reaches the best yields (89.77%) in the presence of 10 wt% Mag-H^+^ at −5 °C. After this time, the copolymerization gradually slows down and the yield becomes almost constant.

### 3.4. Characterization of Nanocomposites (Lim-co-Sty-Mag)

**Figure 11:**
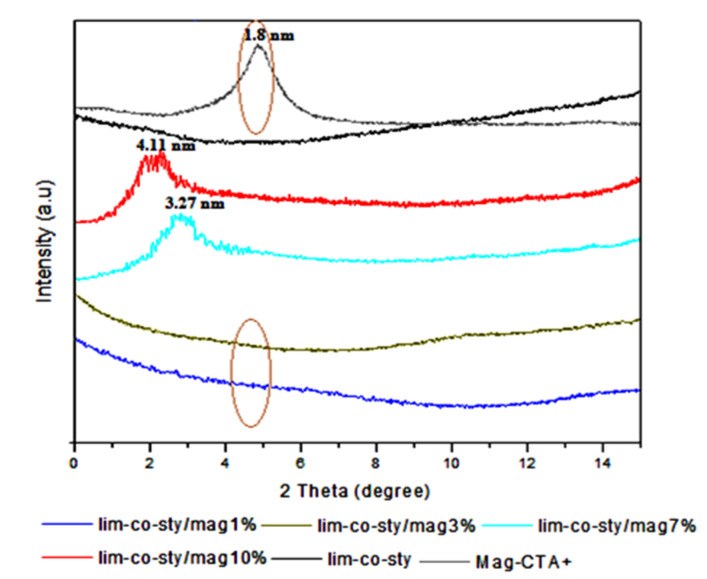
XRD patterns of nanocomposites lim-co-sty/Mag, lim-co-sty and Mag-CTA^+^.

Figure 11 shows the XRD patterns of Mag-CTA^+^, obtained copolymer and nanocomposites. We observed that The XRD pattern of the obtained copolymer (lim-co-sty) presents no sharp peak confirming its amorphous structure. In the case of lim-co-sty/Mag 1% and 3%, the characteristic basal diffraction peak of Mag-CTA^+^ at 2θ = 4.9° was nearly disappeared, confirming the exfoliation of the clay, which explains a good diffusion of lim-co-sty copolymer in the clay galleries. The nanocomposites prepared by (7% and 10%) of Mag-CTA^+^ showed a single peak around 2θ = 2° and 3° corresponding to the interlayer distances d_001_ = 4.15 and 3.3 nm, respectively. The interlayer distance of these nanocomposites was increased more than twice compared to the Mag-CTA^+^, which had an interlayer distance of 1.8 nm. This result confirms that the copolymer was well intercalated between the clay galleries. These results are in agreement with those obtained by Hanène Salmi-Mani et al. [49].

**Figure 12:**
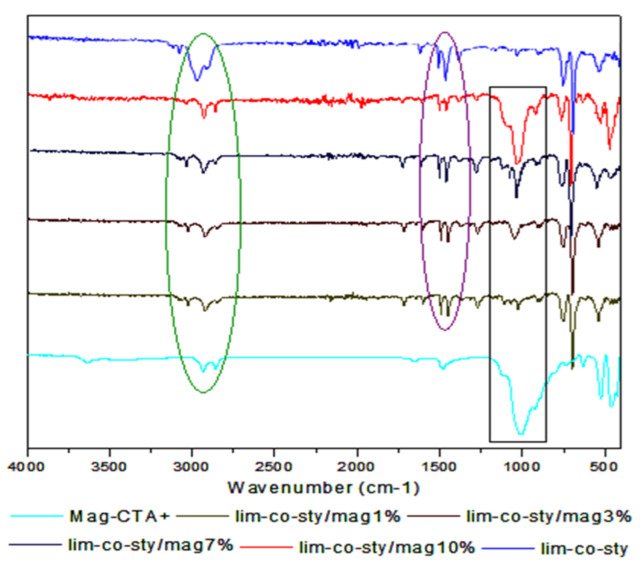
FT-IR spectra of the obtained nanocomposites lim-co-sty/Mag (1%, 3%, 7% and 10%).

The FT-IR spectra of the obtained nanocomposites (lim-co-sty/Mag 1%, 3%, 7% and 10%) are shown in Figure 12. We observed that the obtained nanocomposites are in a good agreement with the pure copolymer structure and have almost the same vibration bands overlapping with the vibration bands of the organo-modified clay (Mag-CTA^+^). The absorption band at 695 cm^−1^ corresponds to the vibration of the benzyl cycle in styrene and the adsorption band at 1600 cm^−1^ corresponds to the double bond C=C in the copolymer were observed in the FT-IR spectra of the obtained nanocomposites. The C-H symmetric and asymmetric stretching of the methyl and methylene group was observed at 2921 and 2867 cm^−1^. Compared with the FT-IR spectrum of the pure copolymer, the spectra of the obtained nanocomposites show the appearance of the intense peak at 1000 cm^−1^ corresponding to the vibration of Si-O of the Mag-CTA^+^. These results show the intercalation of lim-co-sty copolymer in the interlayer montmorillonite gallery.

**Figure 13:**
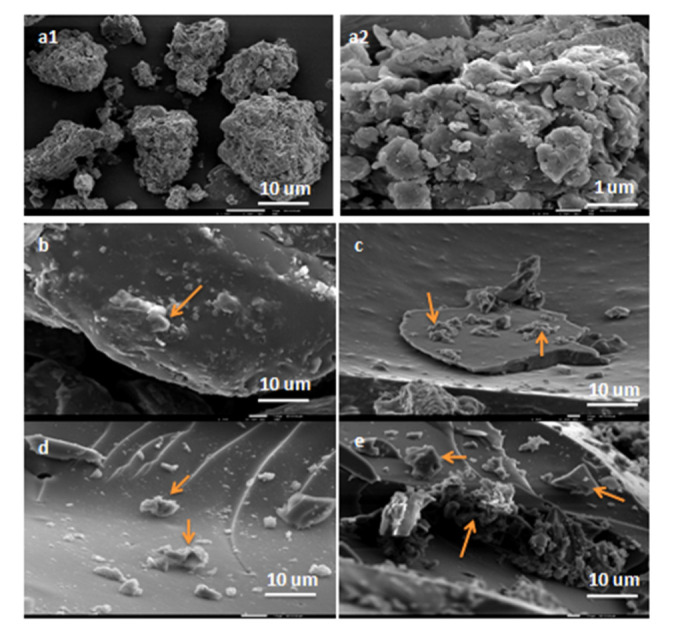
SEM images of Mag-CTA^+^ (**a1**,**a2**), lim-co-sty/Mag 1% (**b**), lim-co-sty/Mag 3% (**c**), lim-co-sty/Mag 7% (**d**) and lim-co-sty/Mag 10% (**e**).

Figure 13 shows the SEM images of the Mag-CTA^+^ and the obtained nanocomposites (lim-co-sty/Mag 1%, 3%, 7% and 10%). Comparing the morphology of Mag-CTA^+^ (Figure 13a1,a2) with lim-co-sty/Mag 7% and 10% nanocomposites (Figure 13d,e), we observed a more organized montmorillonite structure in small particles. In the lim-co-sty/Mag 1% and 3% nanocomposites (Figure 13b,c), the observation of nanocomposites at 10 μm, reveals a formation of separated montmorillonite plate, that is a partial exfoliation, also shows a rougher surface and a covering of the montmorillonite surface by the copolymer.

**Figure 14:**
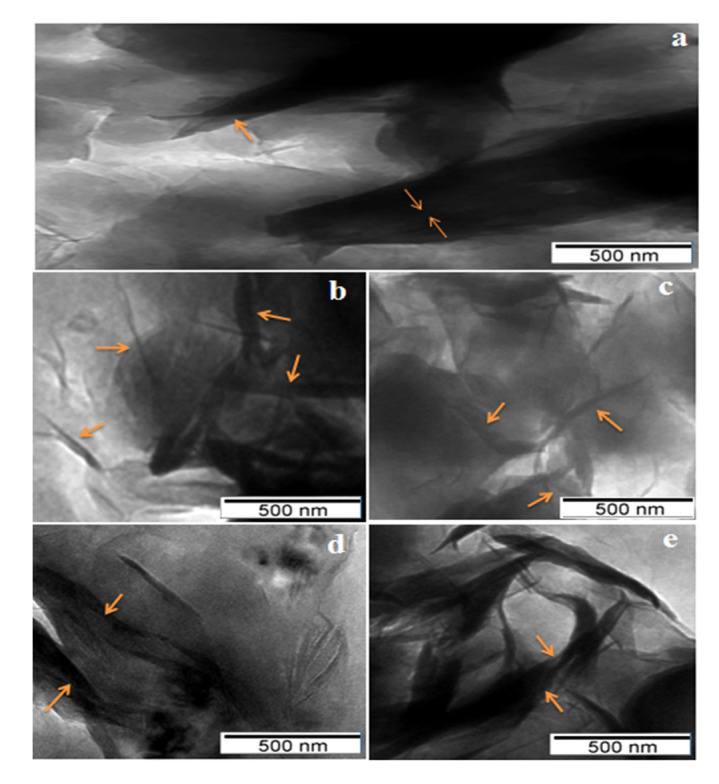
TEM images of Mag-CTA^+^ (**a**), lim-co-sty/Mag 1% (**b**), lim-co-sty/Mag 3% (**c**), lim-co-sty/Mag 7% (**d**) and lim-co-sty/Mag 10% (**e**).

The transmission electron microscopy (TEM) images of Mag-CTA^+^ and the obtained nanocomposites are shown in Figure 14. TEM analysis was used to determine the dispersion of Mag-CTA^+^ in the copolymer matrix and, also, to confirm the results obtained by XRD analysis. For Mag-CTA^+^, it is easy to define the silicate layers by the dark and bright lines. The nanocomposites prepared with 1% and 3% by weight of Mag-CTA^+^ show a partial or total exfoliated structure and the clay nanoparticles are mainly well dispersed in the copolymer matrix. However, the nanocomposites lim-co-sty/Mag 7% and 10% show an intercalated structure of the modified clay. These results confirm the results obtained by XRD analysis.

**Figure 15:**
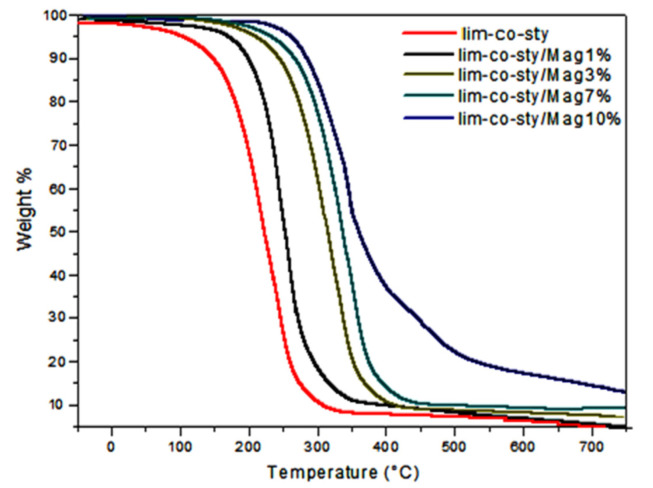
TGA curves of lim-co-sty/Mag nanocomposites and the pure copolymer lim-co-sty.

TGA curves of the obtained nanocomposites and pure copolymer are shown in Figure 15. We observed that all nanocomposites and pure copolymer exhibit a one-step weight loss mechanism. TGA curves show that the Mag-CTA^+^ causes improvement in the thermal stability of the obtained nanocomposites. It can be seen that nanocomposites prepared with 7% and 10% by wt of Mag-CTA^+^ show a high thermal stability up to a degradation temperature about 300 °C, while the degradation temperature of pure copolymer observed at 150 °C, more the nanocomposite is rich in copolymer the more it is degraded quickly. This gain in stability is due, according to previous work [50], to the formation of a protective carbonized layer. The formation of this layer is favored by the fine dispersion of intercalated or exfoliated particles of clay which play an inorganic support role [51]. In general, the degradation temperature of the polymers is increased after the incorporation of exfoliated lamellar silicates [52,53,54], which values these polymers and allows their use at higher temperatures.

## 4. Conclusions

The copolymerization of limonene with styrene was successfully obtained using Mag-H^+^ as green catalyst and provides excellent results. ^1^H-NMR, FTIR and DSC analysis confirm the structure of the obtained copolymer. The copolymerization proceeds via a cationic mechanism due to the presence of intercalated protons in the lamellar structure of Mag-H^+^. According to the study carried out on the operating parameters of the copolymerization reaction we can conclude that the yield achieved is maximum (89.77%) in the presence of 10 wt% of Mag-H^+^ with 6 h of reaction time, wherein the best temperature which promotes copolymerization is −5 °C. The effect of organomodified clay (Mag-CTA^+^), prepared and used with different ratios, on lim-co-sty/Mag nanocomposites properties is also studied. The study shows that the different ratios of the organoclay (Mag-CTA^+^) has an impact in the preparation of nanocomposites copolymer/clay. The FT-IR and XRD results indicate that the nanocomposite prepared with 1% and 3% by weight of Mag-CTA^+^ were exfoliated, and the nanocomposites prepared with 7% and 10% wt of Mag-CTA^+^ were intercalated, leading to an expansion of the interlayer distance between the layers. SEM and TEM analysis confirmed an organization of certain particles, and in other cases a separation in plates made up of montmorillonite layers, this confirms partial or total exfoliation of montmorillonite in the copolymer matrix and formation of the nanocomposites. Thermogravimetric results indicate that the nanocomposites present a higher thermal stability compared with the pure copolymer (T < 300 °C). The objectives of this work are the synthesis of copolymer and nanocomposites out of a green raw material (limonene and clay) by the use of Mag-H^+^ as a catalyst and Mag-CTA^+^ as nano-reinforcing filler. The interesting aspect of clay is the environmentally friendly nature of the reaction because it does not imply the disposal of solvents or metal catalysts.

## Figures and Tables

**Table 1:** Experimental conditions for the preparation of nanocomposites lim-co-sty/Mag.

Samples	Lim-co-Sty	Mag-CTA^+^	Time	Frequency	Yield
Lim-so-sty/Mag 1%	1 g	1% (wt)	3 h	20 KHz	100%
Lim-co-sty/Mag 3%	1 g	3% (wt)	3 h	20 KHz	100%
Lim-co-sty/Mag 7%	1 g	7% (wt)	3 h	20 KHz	100%
Lim-co-sty/Mag 10%	1 g	10% (wt)	3 h	20 KHz	100%

## Data Availability

The data contained in the paper is available from the authors.

## References

[1] Fenouillot F., Rousseau A., Colomines G., Saint-Joup R., Pascault J.P. (2010). Polymers from Renewable 1,4:3,6-Dianhydrohexitols (Isosorbide, Isomannide and Isoidide): A Review. Prog. Polym. Sci..

[2] Lavilla C., Martínez de Ilarduya A., MAlla M.G., García-Martín A., Galbis J.A., Muñoz-Guerra S. (2012). Bio-Based Aromatic Polyesters from a Novel Bicyclic Diol Derived from d-Mannitol. Macromolecules.

[3] Jasinska L., Koning C.E.J. (2010). Waterborne polyesters partially based on renewable resources. J. Polym. Sci. Part A Polym. Chem..

[4] Jansen D.J., Shenvi R.A. (2014). Synthesis of medicinally relevant terpenes: Reducing the cost and time of drug discovery. Future Med. Chem..

[5] Rukel E., Wojcik R., Arlt H. (1976). Cationic Polymerization of α-Pinene Oxide and β-Pinene Oxide by a Unique Oxonium lonCarbenium Ion Sequence. J.Macromol. Sci. Part A.

[6] Sharma S., Srivastava A.K. (2004). Synthesis and characterization of copolymers of limonene with styrene initiated by azobisisobutyronitrile. Eur. Polym. J..

[7] Keszler B., Kennedy J.P. (1992). Synthesis of high molecular weight poly (β-pinene). Adv. Polym. Sci..

[8] Ham G.E. (1964). Penultimate unit effects in terpolymerization. J. Polym Sci. A Polym. Chem..

[9] Derdar H., Belbachir M., Harrane A. (2019). A green synthesis of polylimonene using Maghnite-H^+^, an exchanged montmorillonite clay, as eco-catalyst. Bull. Chem. React. Eng. Catal..

[10] Derdar H., Belbachir M., Hennaoui F., Akeb M., Harrane A. (2018). Green copolymerization of limonene with β-pinene catalyzed by an eco-catalyst Maghnite-H^+^. Polym. Sci. Ser. B.

[11] Doiuchi T., Yamanguchi H., Minoura Y. (1981). Cyclocopolymerization of d-limonene with maleic anhydride. Eur. Polym. J..

[12] Roberts W., Day A. (1950). A Study of the Polymerization of α- and β-Pinene with Friedel- Crafts Type Catalysts. J. Am. Chem. Soc..

[13] Barros M.T., Petrova K.T., Ramos A.M. (2007). Potentially Biodegradable Polymers based on α- or β-Pinene and Sugar Derivatives or Styrene, Obtained under Normal Conditions and Microwave Irradiation. Eur. J. Org. Chem..

[14] Yousif E., Haddad R. (2013). Photodegradation and photostabilization of polymers, especially polystyrene: Review. Springer Plus..

[15] Lai B., Zhou Y., Qin H., Wu C., Pang C., Lian Y., Xu J. (2012). Pretreatment of wastewater from acrylonitrile–butadiene–styrene (ABS) resin. Chem. Eng. J..

[16] Zhang J., Chen H., Zhou Y. (2013). Compatibility of waste rubber powder/polystyrene blends by the addition of styrene grafted styrene butadiene rubber copolymer: Effect on morphology and properties. Polym. Bull..

[17] Devi R.R., Maji T.K. (2012). Chemical modification of simul wood with styrene–acrylonitrile copolymer and organically modified nanoclay. Wood. Sci. Technol..

[18] Mao Z., Zhang J. (2018). Largely improved the low temperature toughness of acrylonitrilestyrene-acrylate (ASA) resin: Fabricated a core-shell structure of two elastomers through the differences of interfacial tensions. Appl. Sur. Sci..

[19] Huang K., Yu S., Li X. (2020). One-pot synthesis of bimetal MOFs as highly efficient catalysts for selective oxidation of styrene. J. Chem. Sci..

[20] Lima M.S., Costa C.S.M.F., Coelho J.F.J., Fonseca A.C., Serra A.C. (2018). Simple strategy toward the substitution of styrene by sobrerol-based monomers in unsaturated polyester resins. Green. Chem..

[21] Kojima Y., Usuki A., Kawasumi M., Okada A., Ukushima Y.F., Kamigaito O. (1993). Mechanical properties of nylon 6-clay hybrid. J. Mat. Res..

[22] Harrane A., Meghabar R., Belbachir M. (2006). In situ polymerization of ε-caprolactone catalyzed by Maghnite TOA to produce poly(ε-caprolactone)/montmorillonite nanocomposites. Des. Monomers Polym..

[23] Zare Y., Fasihi M., Rhee K.Y. (2017). Efficiency of stress transfer between polymer matrix and nanoplatelets in clay/polymer nanocomposites. Appl. Clay. Sci..

[24] Kotal M., Bhowmick A.K. (2015). Polymer nanocomposites from modified clays: Recent advances and challenges. Prog. Polym. Sci..

[25] Mykola S., Olga N., Dmitry M. (2016). The influence of alkylammonium modified clays on the fungal resistance and biodeterioration of epoxy-clay nanocomposites. Int. Biodeterior. Biodegrad..

[26] Bhanvase B.A., Pinjari D.V., Gogate P.R., Sonawane S.H., Pandit A.B. (2011). Process intensification of encapsulation of functionalized CaCO3 nanoparticles using ultrasound assisted emulsion polymerization. Chem. Eng. Processing Process Intensif..

[27] Bhanvase B.A., Pinjari D.V., Gogate P.R., Sonawane S.H., Pandit A.B. (2012). Synthesis of exfoliated poly(styrene-co-methyl methacrylate)/montmorillonite nanocomposite using ultrasound assisted in situ emulsion copolymerization. Chem. Eng. J..

[28] Bhanvase B.A., Pinjari D.V., Gogate P.R., Sonawane S.H., Pandit A.B. (2012). Analysis of semibatch emulsion polymerization: Role of ultrasound and initiator. Ultrason. Sonochem..

[29] Bhanvase B.A., Sonawane S.H., Pinjari D.V., Gogate P.R., Pandit A.B. (2014). Kinetic studies of semibatch emulsion copolymerization of methyl methacrylate and styrene in the presence of high intensity ultrasound and initiator. Chem. Eng. Processing Process Intensif..

[30] Bhanvase B.A., Sonawane S.H. (2014). Ultrasound Assisted In-Situ Emulsion Polymerization for Polymer Nanocomposite: A Review. Chem. Eng. Processing Process Intensif..

[31] Yusof N.S.M., Babgi B., Alghamdi Y., Aksu M., Madhavan J., Ashokkumar M. (2016). Physical and chemical effects of acoustic cavitation in selected ultrasonic cleaning applications. Ultrason. Sonochem..

[32] Cherifi Z., Boukoussa B., Zaoui A., Belbachir M., Meghabar R. (2018). Structural, morphological and thermal properties of nanocomposites poly(GMA)/clay prepared by ultrasound and in-situ polymerization. Ultrason. Sonochem..

[33] Sepúlveda F.A., Rivera F., Loyo C., Canales D., Moreno-Serna V., Benavente R., Rivas L.M., Ulloa M.T., Gil-Castell O., Ribes-Greus A. (2022). Poly (lactic acid)/D-limonene/ZnO bio-nanocomposites with antimicrobial properties. J. Appl. Polym. Sci..

[34] Morère J., Sánchez-Miguel E., Tenorio M.J., Pando C., Cabañas A. (2017). Supercritical fluid preparation of Pt, Ru and Ni/graphene nanocomposites and their application as selective catalysts in the partial hydrogenation of limonene. J. Supercrit. Fluids.

[35] Vaschetti V.M., Eimer G.A., Cánepa A.L., Casuscelli S.G. (2021). Catalytic performance of V-MCM-41 nanocomposites in liquid phase limonene oxidation: Vanadium leaching mitigation. Micropor. Mesopor. Mater..

[36] Derdar H., Mitchell G.R., Mahendra V.S., Beachgoer M., Haoue S., Cherifi Z., Bachari K., Harrane A., Meghabar R. (2020). Green Nanocomposites from Rosin-Limonene Copolymer and Algerian Clay. Polymers.

[37] Elabed Z.O., Kherroub D.E., Derdar H., Belbachir M. (2021). Novel Cationic Polymerization of β-Myrcene Using a Proton Exchanged Clay (Maghnite-H+). Polym. Sci. Ser. B.

[38] Haoue S., Derdar H., Belbachir M., Harrane A. (2020). Polymerization of ethylene glycol dimethacrylate (EGDM), using an Algerian clay as eco-catalyst (maghnite-H^+^ and Maghnite-Na^+^). Bull. Chem. React. Eng. Catal..

[39] Haoue S., Derdar H., Belbachir M., Harrane A. (2020). A New Green Catalyst for Synthesis of bis-Macromonomers of Polyethylene Glycol (PEG). Chemistry.

[40] Derdar H., Meghabar R., Benachour M., Mitchell G.R., Bachari K., Belbachir M., Cherifi Z., Baghdadli M.C., Harrane A. (2021). Polymer-Clay Nanocomposites: Exfoliation and Intercalation of Organophilic Montmorillonite Nanofillers in Styrene–Limonene Copolymer. Polym. Sci. Ser. A.

[41] Cherifi Z., Zaoui A., Boukoussa B., Derdar H., Elabed Z.O., Zeggai F.Z., Meghabar R., Chebout R., Bachari K. (2022). Ultrasound-promoted preparation of cellulose acetate/organophilic clay bio-nanocomposites films by solvent casting method. Polym. Bull..

[42] Derdar H., Mitchell G.R., Cherifi Z., Belbachir M., Benachour M., Meghabar R., Bachari K., Harrane A. (2020). Ultrasound assisted synthesis of polylimonene and organomodified-clay nanocomposites: A structural, morphological and thermal properties. Bull. Chem. React. Eng. Catal..

[43] Cicel B. (1992). Mineralogical composition and distribution of Si, Al, Fe, Mg and Ca in the fine fractions of some Czech and Slovak bentonites. Geol. Carpathica Ser. Clays.

[44] Grenier A., Wendorff H. (2007). Electrospinning: A Fascinating Method for the Preparation of Ultrathin Fibers. Angew. Chem. Int. Ed..

[45] Khenifi A., Zohra B., Kahina B., Houari H., Zoubir D. (2009). Removal of 2,4- DCP from wastewater by CTAB/bentonite using one-step and two-step methods: A comparative study. Chem. Eng. J..

[46] Rieger J. (1996). The glass transition temperature of polystyrene. J. Therm. Anal..

[47] Singh A., Kamal M. (2012). Synthesis and characterization of polylimonene: Polymer of an optically active terpene. J. Appl. Polm. Sci..

[48] Galanos E., Wahlen C., Bytt H.J., Frey H., Floudas G. (2022). Phase Diagram of Tapered Copolymers Based on Isoprene and Styrene. Macromol. Chem. Phys..

[49] Salmi-Mani H., Ait-Touchente Z., Lamouri A., Carbonnier B., Caron J.F., Benzarti K., Chehimi M.M. (2016). Diazonium salt-based photoiniferter as a new efficient pathway to clay–polymer nanocomposites. RSC. Adv..

[50] Bureau M.N., Denault J., Cole K.C., Enright G.D. (2002). The role of crystallinity and reinforcement in the mechanical behavior of polyamide-6/ clay nanocomposites. Polym. Eng. Sci..

[51] Kherroub D.E., Belbachir M., Lamouri S. (2014). Nylon 6/clay nanocomposites prepared with Algerian modified clay (12-maghnite). Res. Chem. Int..

[52] Vaia R.A., Price G., Ruth P.N., Nguyen H.T., Lichtenhan J. (1999). Polymer/layered silicate nanocomposite as high performance ablative materials. Appl. Clays Sci..

[53] Zhu Z.K., Yang Y., Yin J., Wang X.Y., Ke Y.C., Qi Z.N. (1999). Preparation and properties of organosoluble montmorillonite/polyimide hybrid materials. J. Appl. Polym. Sci..

[54] Wang S., Long C., Wang X., Li Q., Qi Z. (1998). Synthesis and properties of silicone rubber/organomontmorillonite hybrid nanocomposites. J. Appl. Polym. Sci..

